# Effects of intraocular lenses with different diopters on chromatic aberrations in human eye models

**DOI:** 10.1186/s12886-016-0184-6

**Published:** 2016-01-11

**Authors:** Hui Song, Xiaoyong Yuan, Xin Tang

**Affiliations:** Tianjin Eye Hospital, Tianjin Key Laboratory of Ophthalmology and Vision Science, Clinical College of Ophthalmology, Tianjin Medical University, No. 4 Gansu Rd, Heping District, Tianjin, 300020 China

**Keywords:** Intraocular lens, Computer simulation, Diopter, Chromatic aberration, Modulation transfer function

## Abstract

**Background:**

In this study, the effects of intraocular lenses (IOLs) with different diopters (D) on chromatic aberration were investigated in human eye models, and the influences of the central thickness of IOLs on chromatic aberration were compared.

**Methods:**

A Liou-Brennan-based IOL eye model was constructed using ZEMAX optical design software. Spherical IOLs with different diopters (AR40e, AMO Company, USA) were implanted; modulation transfer function (MTF) values at 3 mm of pupil diameter and from 0 to out-of-focus blur were collected and graphed.

**Results:**

MTF values, measured at 555 nm of monochromatic light under each spatial frequency, were significantly higher than the values measured at 470 to 650 nm of polychromatic light. The influences of chromatic aberration on MTF values decreased with the increase in IOL diopter when the spatial frequency was ≤12 c/d, while increased effects were observed when the spatial frequency was ≥15 c/d. The MTF values of each IOL eye model were significantly lower than the MTF values of the Liou-Brennan eye models when measured at 555 nm of monochromatic light and at 470 to 650 nm of polychromatic light. The MTF values were also found to be increased with the increase in IOL diopter.

**Conclusion:**

With higher diopters of IOLs, the central thickness increased accordingly, which could have created increased chromatic aberration and decreased the retinal image quality. To improve the postoperative visual quality, IOLs with lower chromatic aberration should be selected for patients with short axial lengths.

## Background

In polychromatic light, the retinal image quality is affected by interactions between monochromatic and chromatic aberrations. Some study had shown that ocular longitudinal chromatic aberration (LCA) was low intersubject variability and the LCA was independence of the presence of high order aberration [1]. Chromatic aberration has attracted increasing attention in intraocular lenses (IOLs) implantation for cataract surgeries. Recent studies have demonstrated that correcting monochromatic aberrations and chromatic aberrations at the same time could improve visual acuity and contrast sensitivity [2–4]. Applegate et al. [5] also demonstrated that correction of spherical aberrations could increase the contrast sensitivity of the retina by 12-fold, and correcting chromatic aberration could increase contrast sensitivity by 5-fold, while correcting both spherical and chromatic aberrations simultaneously could strikingly increase contrast sensitivity by 25-fold.

In Weeber’s study, subjects were implanted with an IOL correcting both LCA and spherical aberration (SA) in one eye and an IOL correcting only SA in the fellow eye. Although this study included a small number of subjects, it showed a tendency for better visual performance in the eyes where both aberrations were corrected [6]. Considering that the LCA causes a substantial defocus over the visible range of about 2 diopters (D), the additional correction of this aberration should further improve visual quality even considering the protective mechanisms [7]. Perez-Merino et al. have reported the chromatic difference of focus in two groups of pseudophakic eyes implanted with IOLs of different materials, and found statistical difference, consistent with Abbe number of IOL materials in the 532–787 nm range [8].

Chromatic aberration is not only associated with Abbe number, which is widely known by researchers, but some other factors, including IOL shapes (thickness and radii of curvature) and the value of the refractive index material, could also affect chromatic aberrations [9]. Investigating the effects of IOLs with different diopters on chromatic aberrations in human eyes could help to further improve patients’ visual quality as well as the design of IOLs [10].

In the present study, Liou-Brennan-based IOL eye models [11] were constructed using ZEMAX optical design software (ZEMAX Development Corporation, Bellevue, WA, USA), and IOLs were implanted to investigate the effects of IOLs with different diopters on the modulation transfer function (MTF) values in monochromatic and polychromatic light.

## Methods

### Construction parameters of the Liou-Brennan eye model

**Fig. 1:**
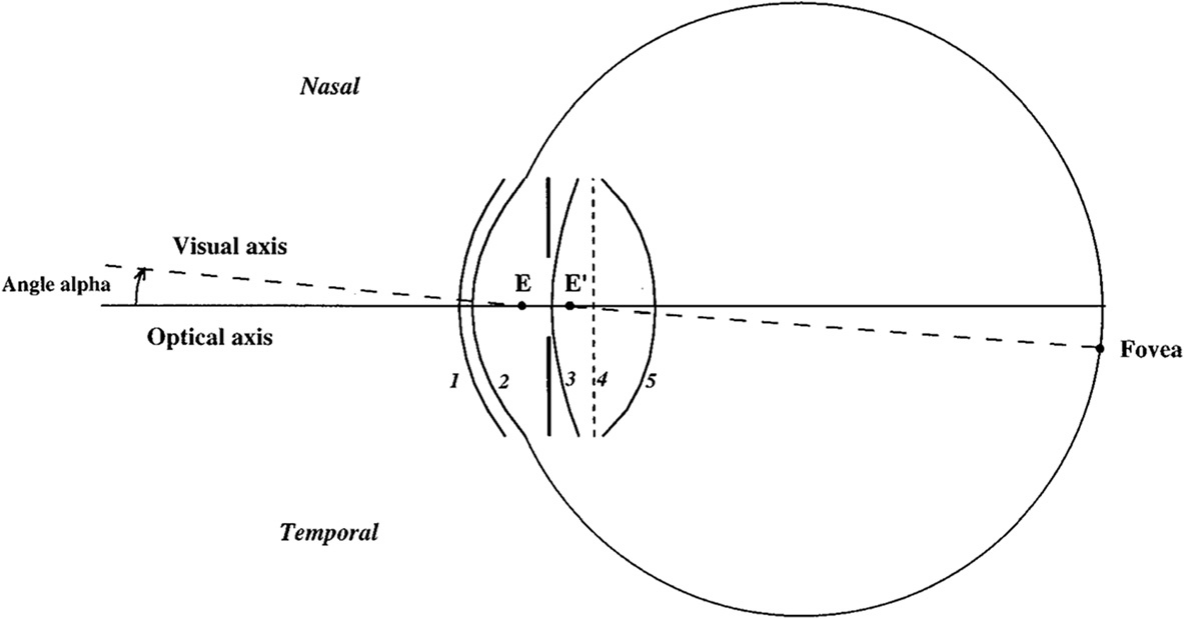
Liou-Brennan eye models

The Liou-Brennan eye model was used as the control (Table 1, Fig. 1), while for the treatment, another eye model, namely an IOL eye model that was constructed according to the process of constructing the control eye model, except for the gradient of the refractive index lens, was replaced by certain IOLs. All the study was approved by the Research Ethics Committee at the Tianjin Eye Hospital.

**Table 1:** Construction parameters of the Liou-Brennan eye model

Surface	Radius of curvature (mm)	Asphericity	Thickness (mm)	Refractive index (555 nm)
1	7.77	−0.18	0.50	1.376
2	6.40	−0.60	3.16	1.336
3(pupil)	12.40	−0.94	1.59	Grad A^a^
4	Infinity	—	2.43	Grad P^a^
5	−8.10	+0.96	16.27	1.336

^a^:Grad A and P represent different formulas for calculating gradient refractive index formula

### Optical parameters of IOLs

To simulate and investigate the retinal image quality of human eye models after implantation of IOLs with different diopters (−10 D, 0 D, 10 D, 20 D, and 30 D), traditional spherical IOLs AR40e, (Abbott Medical Optics, Santa Ana, CA, US) were used in the present study. The optical parameters of these IOLs are listed in Table 2.

**Table 2:** Optical parameters and axial lengths of IOLs with different diopters

Diopters (D)	Refractive index	Curvature radius of the anterior surface (mm)	Central thickness (mm)	Curvature radius of the posterior surface (mm)	Axial length (mm)
+30.00	1.470	6.1951	1.224	−15.7226	22.6597
+20.00	1.470	11.6078	0.977	−15.7226	24.9205
+10.00	1.470	26.7716	0.760	−26.7716	27.7042
0.00	1.470	38.1000	0.533	38.0492	31.3168
−10.00	1.470	50.8000	0.397	10.5918	36.4486

*IOL* intraocular lens

### Construction of the eye models

**Fig. 2:**
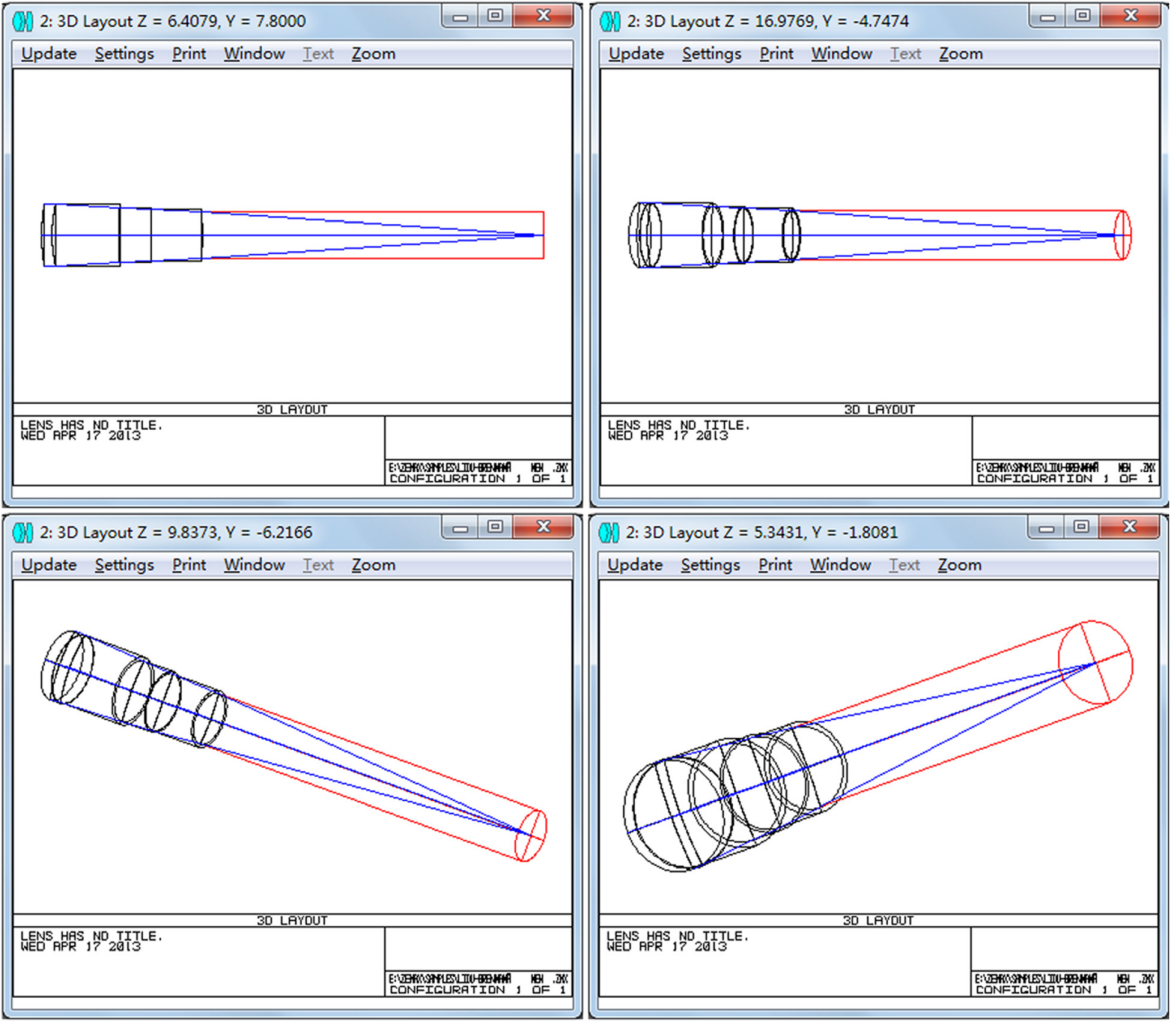
3D stimulated light pathways of Liou-Brennan eye models

The construction of a physical model of the human eyes is based on the anatomical features of humans and on experimental results [12]. The Liou-Brennan eye model has been regarded as the most comprehensive and accurate human model that best fulfills the physiological status of human eyes [13]. The parameters of the Liou-Brennan eye model were entered into the ZEMAX optical design software to construct models of natural and aphakic eyes. Then, IOLs with different diopters were implanted to replace the natural lenses (the posterior surface of the IOL coincided with the posterior surface of the natural lens) to construct the IOL eye model. The vitreous chamber depth was optimized until the out-of-focus blur was 0 (indicating that parallel rays could focus on the retina). The transmission of light through the optical systems was simulated according to the ray-tracing theory (Fig. 2). The axial lengths of the IOLs with different diopters are listed in Table 2.

Modulation transfer function (MTF) is the difference between monochromatic and polychromatic MTF in the same space frequencies. With an IOL diopter increase, the optical zone increases in thickness, resulting in the optical path leading to increased color. A% represents the magnitude of this change. The MTF values at 3 mm of pupil diameter for each of the IOLs were collected with monochromatic (555 nm) and polychromatic (470–650 nm) light under different spatial frequencies and were graphed. Yellow-green light with a wavelength of 555 nm was chosen as the monochromatic light in the present study, while for polychromatic light, we chose light with wavelengths ranging from 470 to 650 nm (including monochromatic light with wavelengths of 470, 510, 555, 610, and 650 nm and with weights of 0.091, 0.503, 1, 0.503, and 0.107, respectively). All of the MTF values were collected using the ZEMAX Optical Design Program.

### Comparison of the MTF curves

Origin software, version 7.0, was used for the data processing and graphing. The trends and difference were compared among the MTF curves that were constructed with the data collected under same conditions. Student’s *t* test was used to compare the differences between the 2 groups. One-way ANOVA test was used to make comparisons between multiple groups (>3 groups), and the Dunnett test was further used to compare different IOLs groups with the Liou group. Statistical significance was set at *p* < 0.05.

## Results

### Comparisons of the MTF values of the IOL eye model under monochromatic and polychromatic light

**Fig. 3:**
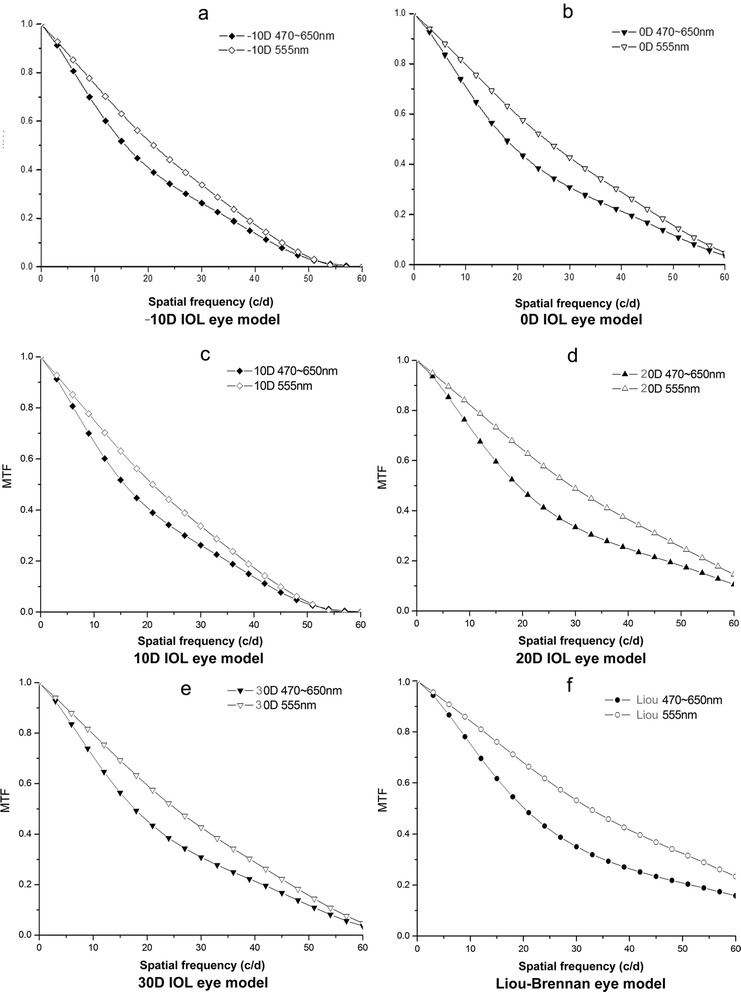
Modulation transfer function (MTF) curves of the IOLs from −10 diopters (D) to 30 D and Liou-Brennan eye models under monochromatic and polychromatic light. **a**. -10 D IOL eye model, **b**. 0 D IOL eye model, **c**. 10 D IOL eye model, **d**. 20 D IOL eye model, **e**. 30 D IOL eye model, **f**. Liou-Brennan eye model

MTF values at 3 mm of pupil diameter, measured at 555 nm of monochromatic light under each spatial frequency (ranging from 0 to 60 c/d), were significantly higher than the values at 470–650 nm of polychromatic light (Fig. 3a–f). In addition, the differences between the MTF values that were measured under monochromatic (555 nm) and polychromatic (470–650 nm) light increased with the IOL diopter (i.e., the central thickness of the IOLs increased with the diopters, and the axial length decreased accordingly) (Fig. 3a–e). We also found that the A-value increased with the spatial frequencies (SFs), indicating the increased influence of chromatic aberration on MTF values (Table 3). In contrast, the A-value decreased (indicating that the influence of chromatic aberration on MTF values decreased) with the increase of IOL diopters when the MTF values were measured at 3 mm of pupil diameter with spatial frequencies ≤12 c/d. However, the A-value was found to be increased with the IOL diopters when the spatial frequencies were ≥15 c/d (Table 3).

**Table 3:** A-value of the IOL eye models with different diopters at 3 mm of the pupil diameter

SF	30D	20D	10D	0D	−10D
(c/d)					
3	1.241	1.285	1.353	1.449	1.595
6	4.495	4.595	4.752	4.991	5.344
9	8.951	9.077	9.270	9.556	9.923
12	13.909	14.019	14.128	14.271	14.341
15	19.031	18.892	18.768	18.548	17.954
24	31.353	30.192	28.620	26.333	22.675
33	37.830	35.336	32.098	27.644	21.559
42	40.159	36.569	31.629	25.449	21.245
51	40.562	35.455	28.944	24.503	11.747
60	39.331	32.501	27.721	23.957	——

*IOL* intraocular lens, *SF* spatial frequencies, *MTF* modulation transfer function, *D* diopter, A value = ΔMTF/MTF_1_; ΔMTF = MTF_1_-MTF_2_; MTF_1_ represents the MTF values measured under 555 nm of monochromatic light, MTF_2_ represents the MTF values measured under 470–650 nm of polychromatic light

### Comparisons of the MTF values between the IOL and Liou-Brennan eye models under monochromatic and polychromatic light

**Fig. 4:**
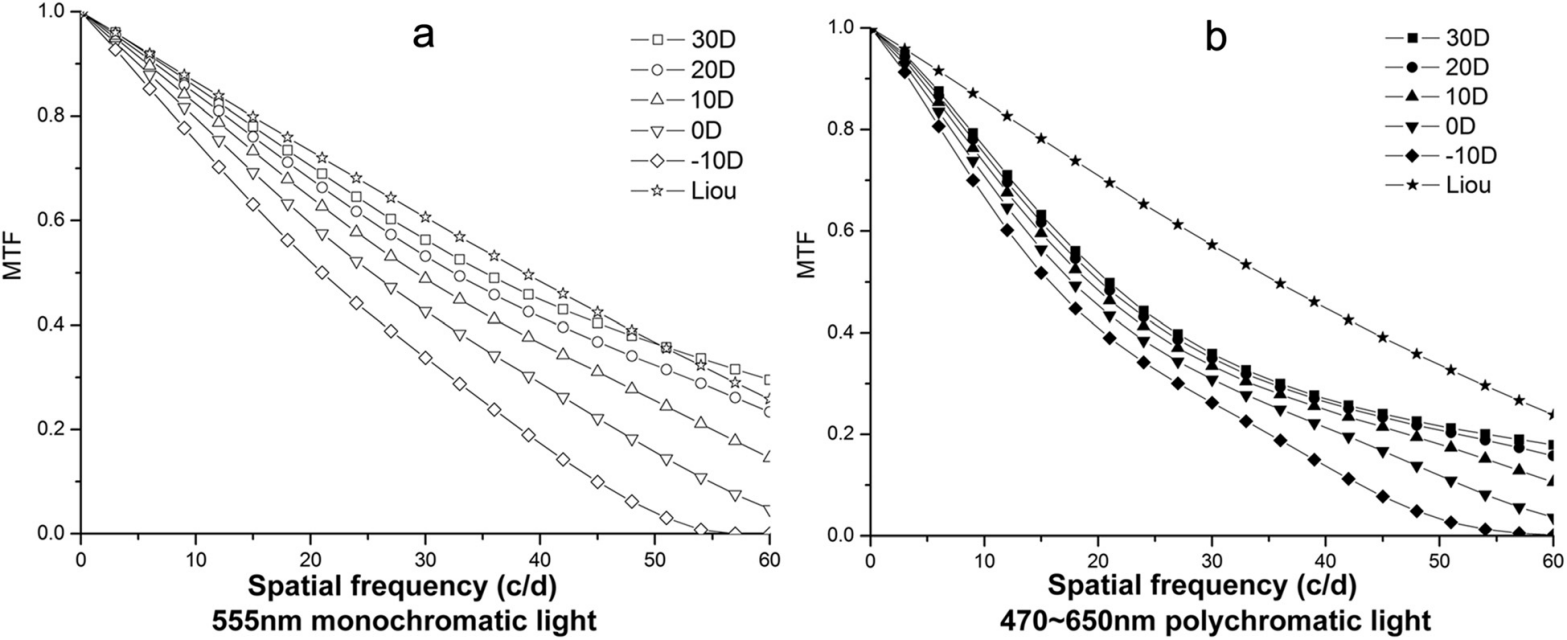
Modulation transfer function (MTF) curves of the IOL from −10 diopters (D) to 30 D and Liou-Brennan eye models under monochromatic and polychromatic light. **a**. 555 nm monochromatic light, **b**. 470–650 nm polychromatic light

The MTF values of most of the IOL eye models were significantly lower than those of the Liou-Brennan eye models when measured under monochromatic light (555 nm), with pupil diameter of 3 mm and spatial frequencies between 0 and 60 c/d, and F values from 10, 20, 30, 40, 50, and 60 c/d were 3.96, 14.35, 29.07, 37.36, 51.34, 69.39 with *p* < 0.05 respectively. However, the MTF value of the IOL eye model with 30 D was slightly higher than the MTF value of the Liou-Brennan eye model when measured with the spatial frequencies ≥50 c/d. The MTF values of the IOL eye models increased with the IOL diopter (Fig. 4a). When measured under polychromatic light (470–650 nm), the MTF values of the IOL eye models were significantly lower than those of the Liou-Brennan eye models, with pupil diameter of 3 mm and spatial frequencies between 0 and 60 c/d with F values 3.38, 8.91, 17.15, 28.69, 47.45, and 64.65 (*p* < 0.05), but the differences between the IOL and Liou-Brennan eye models were substantially less than those under monochromatic light (555 nm). We also observed that the MTF values increased with IOL diopters, as measured under monochromatic light (Fig. 4b).

## Discussion

### Parameters of IOL eye model

The pupil plays an important role in optical imaging [14]. Studies [3, 4, 15, 16] have demonstrated that correcting spherical and chromatic aberrations simultaneously could increase visual acuity and contrast sensitivity under white light as well as provide better visual quality with a broader range of pupil diameters. Decreased visual quality was closely associated with increased spherical aberration of IOLs after the implantation of traditional spherical IOLs [17]; however, a diameter of the pupil of 3 mm is very close to the pupil diameter in diffraction-limited systems (pupil diameter less than 2.5 mm), indicating that the influence from chromatic aberration is even greater than other factors, compared with eyes with their own optical aberrations. Campbell and Gubisch reported that whereas for 4 mm pupils the effect of spherical and chromatic aberration was approximately equal, monochromatic aberrations had a larger effect for large pupils [18]. In the present study, the differences between the MTF values for pupils with diameters less than 3 mm that were measured under monochromatic and polychromatic light were found to increase with diopters, which could have been caused mainly by chromatic aberration. The IOL material and its unique dependency between refractive index and wavelength are of critical importance for the optical performance of IOLs in polychromatic light [19]. As chromatic aberration is associated with the material of the IOL, in the present study, only AR40e IOLs were chosen because they were the same type and were designed with the same materials.

Retinal image quality in the human eyes is dependent on the features of neural pathways. Studies have demonstrated that the cut-off spatial frequency is approximately 60 c/d under photopic conditions [20]; however, when the spatial frequency was increased to 32 c/d, some healthy adults could not detect the gratings, suggesting that they had very low contrast sensitivity [21]. Qu et al. also demonstrated that MTF could be used to reflect the contrast sensitivity of the optical system of the eyes [22]. The MTF values of the eyes of healthy adults are very low at a spatial frequency of 32 c/d and thus could provide very limited value for clinical investigations. Lower spatial frequencies could affect outline identification more than higher spatial frequencies, and most of the information that is received by the eyes is from low spatial frequencies [23]. Because excellent retinal image contrast is also required under low spatial frequencies, in addition to higher cut-off spatial frequencies [24], 0–60 c/d of the spatial frequencies were chosen for the investigation in the present study.

The retina is most sensitive to yellow light with a wavelength of 555 nm under photopic conditions [25]; while for polychromatic light, we chose light with wavelengths ranging from 470 to 650 nm (including monochromatic light with wavelengths of 470, 510, 555, 610, and 650 nm and with weight of 0.091, 0.503, 1, 0.503, and 0.107, respectively). The MTF values were measured under monochromatic and polychromatic light and were compared to investigate the effects of chromatic aberration on retinal image qualities.

### Visual qualities of the IOL eye models

In the present study, we investigated the effects of IOLs on the chromatic aberrations of human eyes with different refraction states. The findings demonstrated that the MTF values were significantly lower when measured under polychromatic light than when measured under monochromatic light for each of the IOLs, without influences from other factors, suggesting that chromatic aberrations could reduce retinal image quality. Polychromatic MTF contains information on both monochromatic and chromatic aberrations [26]. While the monochromatic MTF of the eye clearly exceeds the polychromatic MTF and there is evidence that, in the absence of both chromatic and monochromatic aberrations, visual performance exceeds that with noncorrected chromatic aberrations [8, 27, 28]. In addition, we also found that the difference between the MTF values that were measured under monochromatic and polychromatic light increased with the diopters of the IOLs (indicating that the central thickness of the IOLs increased), suggesting the increased influences of chromatic aberrations on retinal image quality. The findings of the present study also suggested that shorter axial length required IOLs with higher diopters, which could result in higher chromatic aberrations. Therefore, for patients with short axial length who require IOLs with high diopters, IOLs with low chromatic aberration should be selected to improve the postoperative visual quality.

However, with the increase in IOL diopters, the influence of chromatic aberrations on the MTF values decreased when the spatial frequencies were ≤12 c/d for pupils with diameters of 3 mm, which could also reflect the effects of chromatic aberrations on the retinal image quality decreasing at medium or low spatial frequency when IOL with higher central thickness was used. Generally, most of the information that is received by the eyes is from low spatial frequencies, and lower spatial frequencies could affect outline identification more than higher spatial frequencies [23]. In another study performed by Nio et al., the findings showed that the contrast sensitivity of normal human eyes peaked at medium and low spatial frequencies (4–8 c/d) [21]. Therefore, for elderly patients undergoing cataract extraction and IOL implantation, IOLs with higher diopters could reduce the effects of chromatic aberrations on retinal image quality while identifying the outlines of objects, whereas IOLs with lower diopters could increase the effects.

The findings of the present study demonstrated that the MTF values of all of the IOL eye models were lower than those of the Liou-Brennan eye models, under either monochromatic or polychromatic light, suggesting that the implantation of IOLs could reduce the retinal image quality of human eyes [8, 29], although the difference was not that significant for the “common” implanted IOL-powers (from 15 D to 25 D). The differences between the MTF values in the IOL eye models and the Liou-Brennan eye models were not as high under polychromatic light than under monochromatic light (555 nm), suggesting that the introduction of chromatic aberrations could further decrease retinal image quality. Interestingly, we found that the MTF values increased with the IOL diopters, either under 555 nm of monochromatic light or under polychromatic light, which could have been caused by the interactions of monochromatic aberrations and chromatic aberrations in the eye models. In addition, the axial length of the eye model could change after the implantation of IOLs, as well as optimization of optical aberrations and out-of-focus images, which could in turn affect the MTF values. Moreover, with the increase in diopters of the IOLs, the central thickness also increased. Because the thicknesses of IOLs with higher diopters are closer those in the Liou-Brennan eye models (4.02 mm) with the increased IOL diopters, the MTF values of the IOL eye models could also approach the MTF values of the Liou-Brennan eye models. However, no studies have investigated the detailed mechanisms, and further studies are warranted to validate the above-mentioned theories.

## Conclusion

The present study showed that IOLs with different diopters affected chromatic aberrations of the eye when the diameter of the pupil was 3 mm. The chromatic aberrations increased when IOLs with higher diopters (which also indicates higher central thickness) were implanted, thus reducing retinal image quality. The implantation of IOLs with relative high diopters for patients with less axial depth created relatively high chromatic aberrations, which could influence the visual quality of the patients. Interestingly, the increase in IOL diopters was mirrored by the decrease in the influence of the chromatic aberrations on visual quality at medium or low spatial frequencies. However, contradictory results were found at high spatial frequencies. The visual quality of the IOL eye model further decreased due to the effects of chromatic aberrations. The findings of the present study provided evidence for the designing of IOLs in the near future.

## Abbreviations

IOLs: intraocular lenses
D: diopter
MTF: modulation transfer function
SF: spatial frequencies
LCA: longitudinal chromatic aberration
SA: spherical aberration
